# Strata behaviour and stability control of the automatic roadway formation by roof cutting below a fault influenced longwall goaf

**DOI:** 10.1038/s41598-022-20810-7

**Published:** 2022-09-30

**Authors:** Yubing Gao, Qiukai Gai, Kedong Zhang, Qiang Fu, Xingxing Zhang

**Affiliations:** 1 State Key Laboratory for Geomechanics and Deep Underground Engineering, China University of Mining and Technology (Beijing), Beijing, 100083 China; 2 School of Mechanics and Civil Engineering, China University of Mining and Technology (Beijing), Beijing, 100083 China; 3 Institute for Deep Underground Science and Engineering, China University of Mining and Technology (Beijing), Beijing, 100083 China

**Keywords:** Civil engineering, Natural hazards

## Abstract

Automatic roadway formation by roof cutting (ARFRC) is a novel nonpillar mining method that has the potential to dramatically increase coal recovery while reducing the roadway excavation ratio. When this method is used below a fault influenced longwall goaf, large deformation and support failure occur in the roadway using conventional roadway formation techniques. In the study, the ARFRC method was tested in the Liliu mining area of China, which is characterized by goafs and faults. Field experiments and numerical modelling were used to evaluate the stability of the roadway by analysing the behaviour of overlying strata under the special geological condition. The results show that the surroundings of the formed roadway were greatly affected by the fault and the overlying coal pillar in the goaf. In the fault- and coal pillar-affected areas, the loads on the roadway roof increased by approximately 35% and 15%, respectively. According to the strata behaviour of the formed roadway surroundings, targeted support techniques for ARFRC were proposed, and the reliability of the support techniques were demonstrated by field practice.

## Introduction

**Figure 1:**
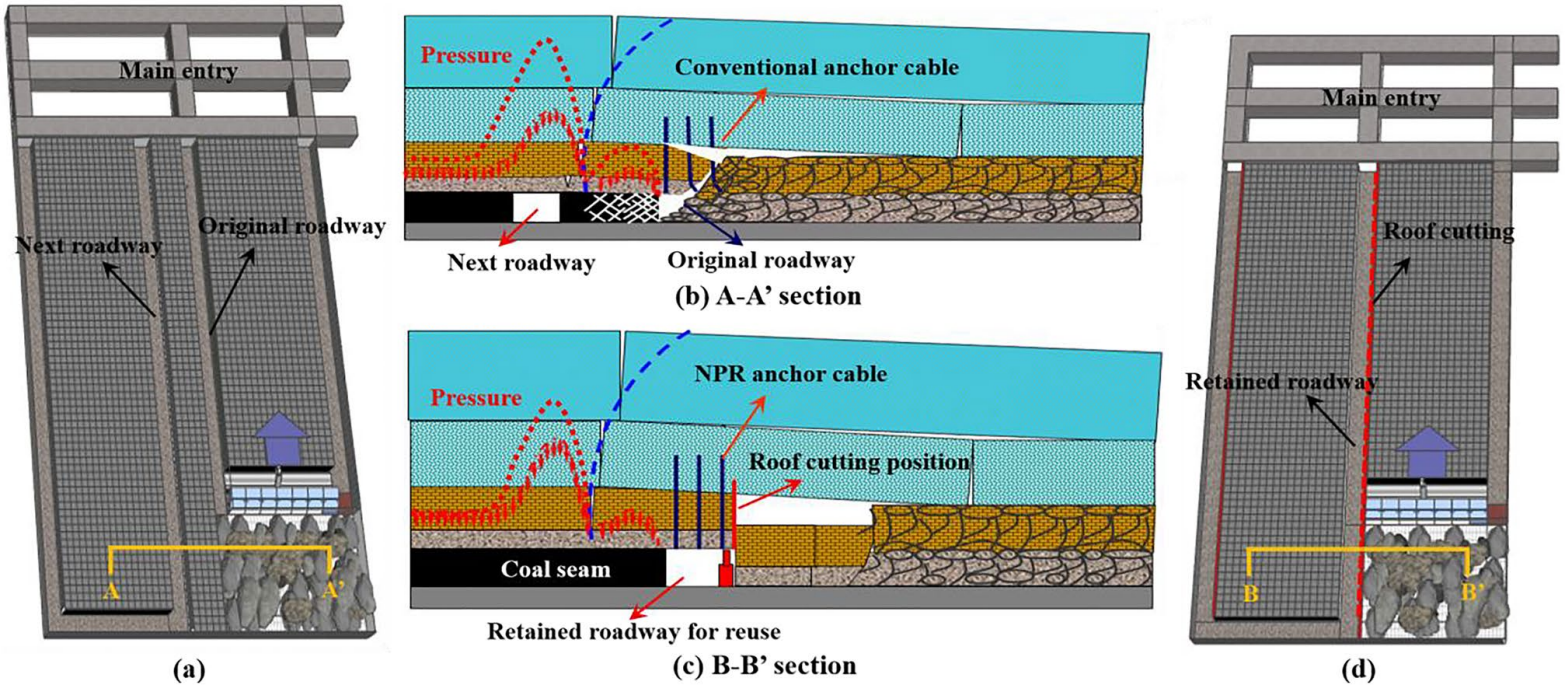
Principle of the conventional longwall mining system and the ARFRC method.

Longwall mining has been a main type of underground coal mining since the early 1970s due to its high mechanization, high productivity and low cost. In a typical longwall mining system, a mining area is divided into multiple working panels. Two roadways are excavated on both sides of a working panel for coal transportation, material transportation and ventilation, while a coal pillar remains between two adjacent panels, as shown in Fig. 1a. There are three problems associated with this kind of mining layout system^1^. First, the remaining coal pillar cannot be mined, and coal resource waste is unavoidable. Second, the roadway drivage ratio is high. One mining panel needs two roadways which are excavated in advance. Third, stress concentration frequently occurs on the coal pillar, which may cause underground disasters such as coal bursts, as shown in Fig. 1b. Mining layouts without coal pillars have been studied since the 1950s, and one commonly used method to achieve a layout without coal pillar is to reserve the roadway by supporting the roof beside the roadway with artificial filling materials^2^. In the early twenty-first century, an improved nonpillar mining method that used roof cutting for automatic roadway formation was proposed^3–5^. In this method, one roadway of the current panel is formed with key technologies such as Negative Poisson's Ratio (NPR) anchor cable support and directional roof cutting, and the formed roadway is then used for the adjacent mining panel, as shown in Fig. 1c and d. In this way, the coal pillar can be completely eliminated in the mining area, and the roadway excavation is reduced by fifty percent.

The ARFRC method has been widely applied in China since 2009. Initially, the applied geological conditions of the method were limited to be simple^6,7^. As its popularity increases, the applied geological conditions have been constantly extended, such as thick coal seam^8^, large buried depth^9^ and large dip angle conditions^10^. In this process, many studies have been conducted on overburden strata movement^11–14^, mine pressure distribution laws^15–17^ and surrounding rock control technology of the formed roadway^18–20^. It was found that the geological condition plays an important role in the roadway formation. Faults are tectonic fractures in the earth’s crust along which one side of a fault plane moves horizontally, vertically, or diagonally relative to the other. Faults commonly encounter geological structures during coal seam mining or roadway excavation. The rocks near the fault structure often have poor self-stabilizing abilities^21,22^. Many studies have been performed on fault activation^23,24^ and fault-induced disasters^25,26^. Studies show that the fault has great influence on the stability of the roadway^27–30^. Although the ARFRC method was applied in different geological conditions, investigations of ARFRC under fault-affected geological conditions are lacking. In our study, the strata behaviour of the roadway surroundings formed by roof cutting below a fault influenced longwall goaf was investigated by discrete element simulations and field experiments. The stress and displacement of the roadway surroundings were analysed to evaluate the stability of the formed roadway. Control techniques were proposed based on the behaviour of the roadway surroundings. The results have guiding significance for the application of the novel nonpillar mining method under complicated geological conditions.

## Engineering overview

### Engineering site

Coal resources are abundant in China, but coking coal resources are relatively small. The Liliu mining area is one of the most important areas in China for producing coking coal. To avoid wasting coal resources, the Xiashanmao coal mine, which is a typical coal mine in the Liliu mining area, began to implement the ARFRC method.

**Figure 2:**
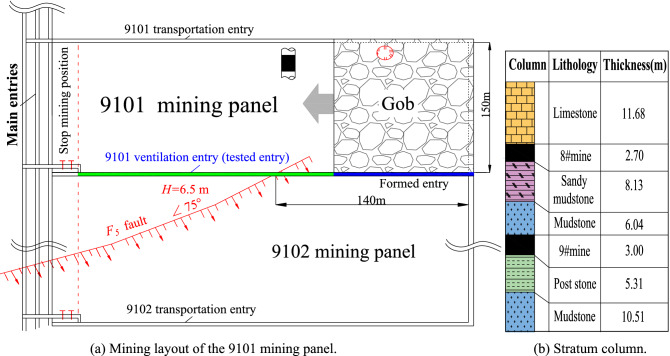
Mining layout of the 9101 longwall panel in the Xiashanmao coal mine.

The ARFRC method was tested on a 9101 mining panel with a mining length of 457 m and burial depth of 205 m. The 9# coal seam was mined from the upper Carboniferous Taiyuan Formation. The thickness of the coal seam varied from 3.0 m to 3.5 m, and the dip angle varied from 2 to 4 degrees. The coal seam was mined using a fully mechanized longwall mining method, and the roof strata was allowed to cave during the mining process. As shown in Fig. 2a and b, the ventilation roadway of the 9101 mining panel was the test roadway, with a width of 3.8 m and height of 3.1 m. The test roadway was formed by the ARFRC method and reused for the 9102 mining panel. Thus, the coal pillar between the 9101 and 9102 mining panels could be mined out.

### Geological characteristics

**Figure 3:**
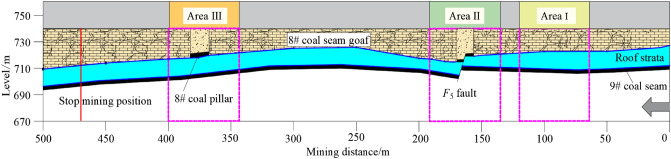
Geological section of the test area.

The study site had typical geological characteristics. First, the formed roadway was located beneath a goaf. In the Liliu mining area, the 8# and 9# coal seams are the two most common mining coal seams. The 9# coal seam was located beneath the 8# coal seam, and the distance between the two seams was small. After the 8# coal seam was mined, the 9# coal seam was mined. The geological section is shown in Fig. 3. The average distance between the 8# coal seam floor and the 9# coal seam roof was 10 m, and the rock stratum between the two coal seams was mainly composed of mudstone. The 8# coal seam panel was mined from south to north. When the coal seam of the panels was mined out, a series of coal pillars with a width of 15 m were left above the 9# coal seam. The 9# coal seam was mined from east to west, considering the overall layout of the mining area. Mining and roadway formation are conducted simultaneously. Therefore, the 9101 mining panel and the formed roadway must cross the gob of the coal pillars.

The second typical feature was that the formed roadway crossed a normal fault during the mining process. The slip distance between the hanging wall and the footwall was 6.5 m, and the dip of the fault was 60 degrees. Because the fault intersected the roadway diagonally, the influence area of the fault was large, approximately 60 m. As the working face advanced, the formed roadway first approached the fault, then crossed the fault, and finally moved away from the fault. Due to the existence of the normal fault, the strata behaviour of the formed roadway surroundings differs considerably from that under normal geological conditions.

**Figure 4:**
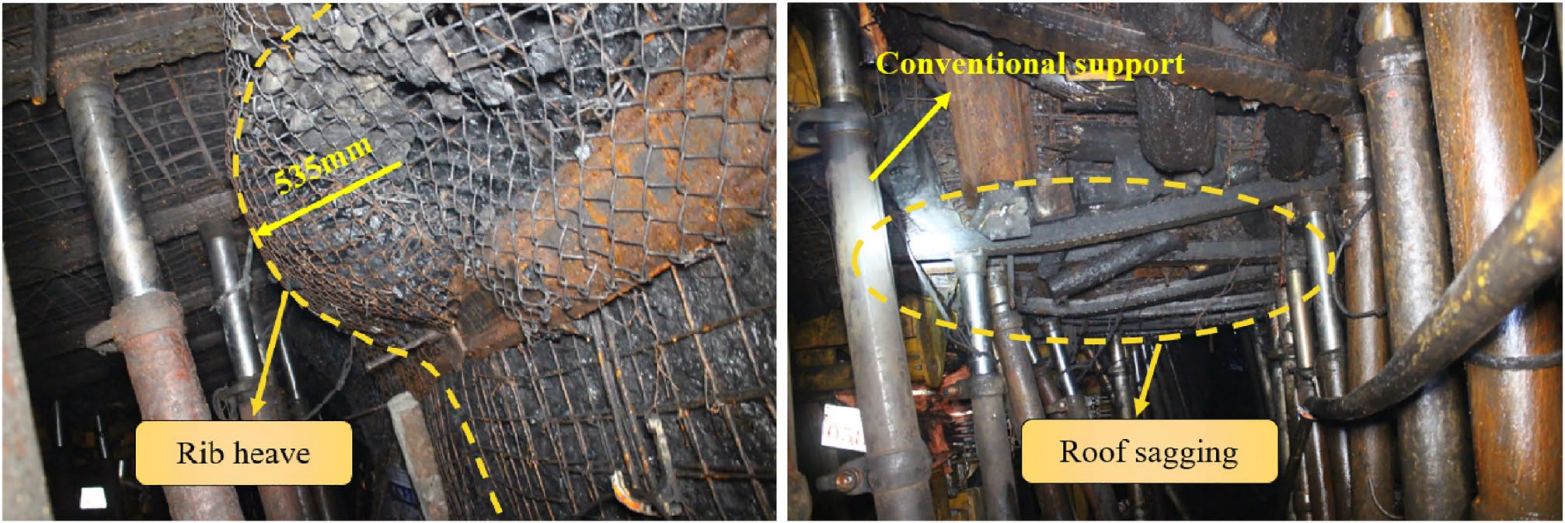
Large deformation of the roadway surroundings and failure of the support.

Because the roadway formation process occurs below the mined-out area of the 8# coal seam and the distance between the 8# and 9# coal seams is small. Under the influence of fault influenced longwall goafs, conventional supports show obvious limitations. As shown in Fig. 4, in the initial section of the roadway below a fault influenced longwall goaf, three rows of single hydraulic props with π beams were used to support the roof. Single props skew seriously, leading to large deformations in the roof and coal rib. The maximum deformations of rib heave and roof sagging reached 535mm and 1020mm, respectively. In view of this, it’s essential to analyse the behaviour of the roadway surroundings and present targeted control techniques.

## Methodology

### Experimental study

#### Experimental process

**Figure 5:**
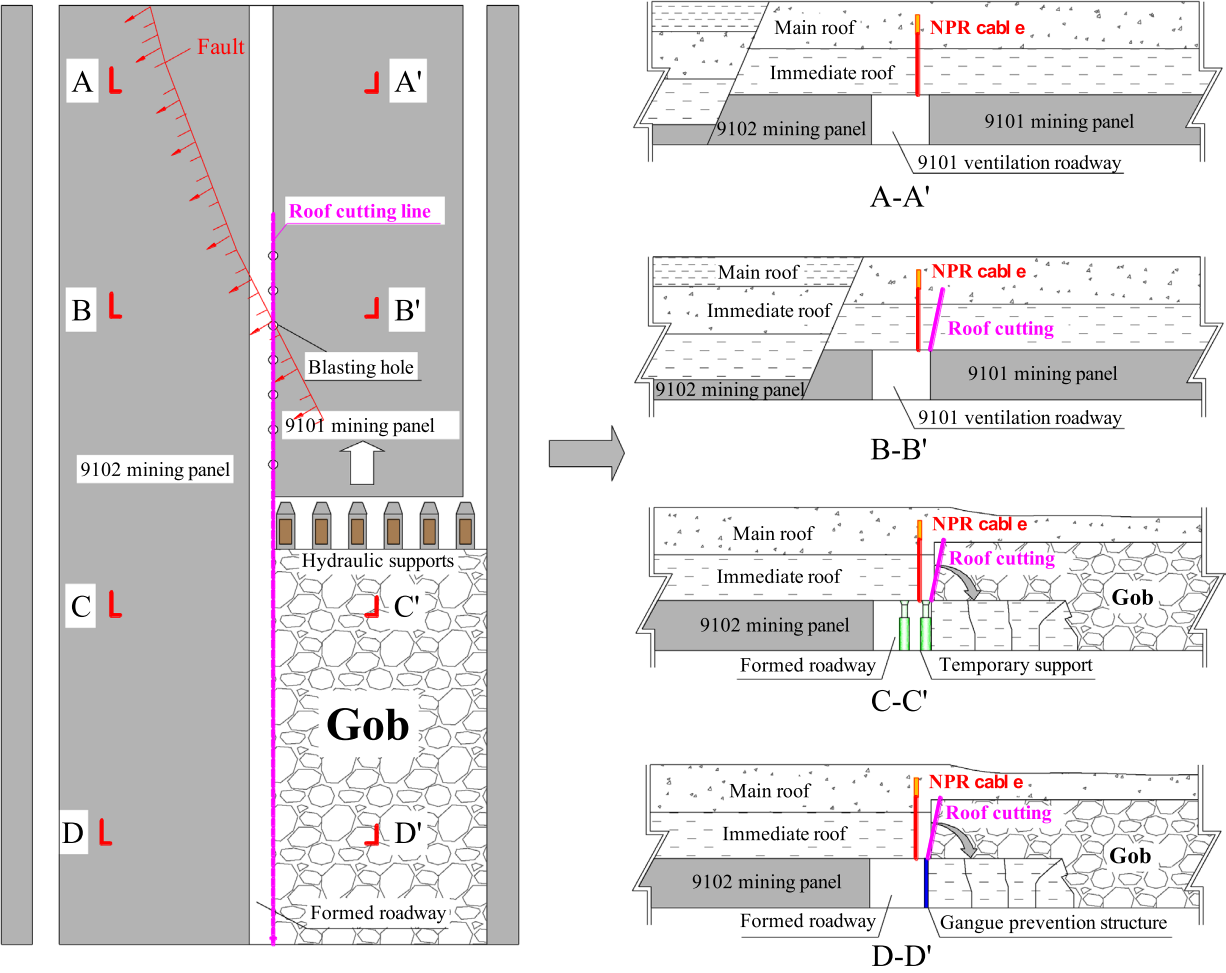
Experimental process of the ARFRC method in the 9101 ventilation roadway.

To explore the failure mechanism of the automatic roadway formation by roof cutting under special geological conditions, the field experiment was conducted on the 9101 ventilation roadway. The plan and section of the mining panel are shown in Fig. 5. The specific test process was as follows.

*Step I:* Before the working face was mined, the roadway roof was reinforced by NPR anchor cables in front of the active working face. The length and diameter of the cable were 9.0 m and 21.8 mm, respectively. The distance between the two cables was 1.0 m.

*Step II:* After the NPR anchor cables were installed, roof cutting was performed on the side of the roadway roof. Blast holes with a depth of 7.5 m and diameter of 50 mm were drilled, and an energy-gathering device containing 2800 g explosives was installed in each hole. After blasting, a directional roof cutting plane was generated.

*Step III:* Based on the geological characteristics, partitioning and marking were performed on the formed roadway. The behaviour of the roadway surroundings was compared in three areas. As shown in Fig. 3, the first typical area was below the goaf of the 8# coal seam (Area I), the second typical area was the area affected by the fault (Area II), and the third area was below the coal pillar of the 8# coal seam (Area III). Instruments were installed in these areas to monitor the roof strata pressure and displacement.

*Step IV:* After the roadway was formed, temporary support equipment was used to support the roof. During the mining process, the behaviour of the roadway surroundings was recorded to investigate the influence of the geological structure on the stability of the roadway surroundings.

#### Methodology and instruments

**Figure 6:**
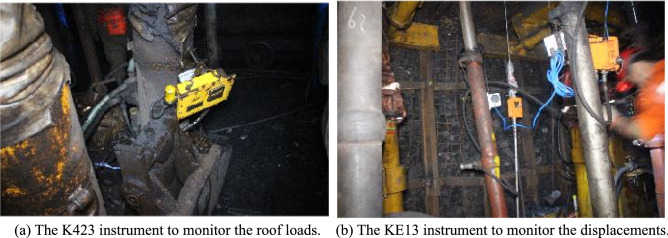
Monitoring instruments for the roadway surrounding behaviours in the mining face and formed roadway.

During the field experiment, the loads of the roof strata on the support equipment and the deformation of the roadway surroundings were monitored. The pressure on the hydraulic support in the active working face can reflect the movement of the overlying strata. A K423 hydraulic monitoring instrument was used to monitor the hydraulic pressure in the active mining face (see Fig. 6a). This instrument can collect data online in real time and sends data every five seconds. The gangues at the gob experienced caving, compacting and stabilizing processes, and the deformation of the roadway roof during these processes can reflect the stability of the formed roadway. In the field test, a KE13 instrument for roof-to-floor convergence was installed in the formed roadway to monitor the displacement of the roadway surroundings, as shown in Fig. 6b.

### Numerical modelling

Rock strata are discontinuous geological media that are often cut by numerous beddings and joints. Because it is difficult to directly measure the behaviour of a fault in the field, discrete element method (DEM) numerical simulations were used to investigate the behaviour of the roadway surroundings. In the DEM numerical simulation, the rock mass was assumed to be composed of discrete blocks and joints. The blocks can be moved, rotated, and deformed, and the joint surfaces can be separated or slipped. During the simulation process, separation, sliding, and rotation behaviours of the rock block can be realized, and the large deformation characteristics of the jointed rock mass can be simulated more realistically.

#### Constitutive model

1. Deformable block model

**Figure 7:**
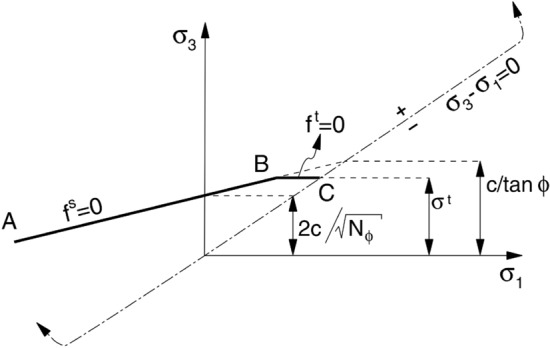
Constitutive model for the block material.

Considering that the rock material is prone to failure due to tensile stress, the Mohr–Coulomb elastoplastic constitutive model, which considers tensile strength, was used for the deformable block material in the simulation. The constitutive relation is illustrated in Fig. 7. From point B to C, the failure envelope is defined by the tensile yield function:

1 $$f^{t} = \sigma_{t} - \sigma_{3}$$

where $f^{t}$ is the shear yield function; $\sigma_{t}$ is the tensile strength of the rock material; and $\sigma_{3}$ is the minimum principal stress.

From point A to B, the failure envelope is defined by the Mohr–Coulomb yield function:

2 $$f^{s} = \sigma_{1} - \sigma_{3} N_{\phi } + 2c\sqrt {N_{\phi } }$$

where $f^{s}$ is the shear yield function, $\sigma_{1}$ is the maximum principal stress, and $N_{\phi }$ is a function calculated by the internal friction angle.

(2) Contact model

**Figure 8:**
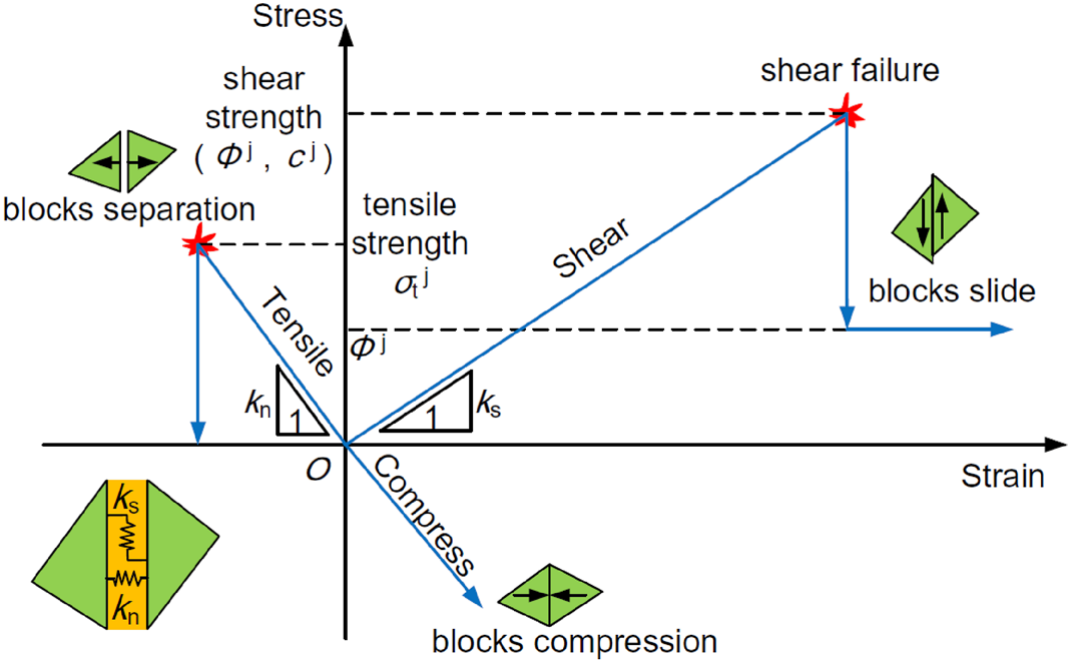
Constitutive behaviour of the contact.

In the joint constitutive model, a Coulomb slip joint model with surfaces in contact was adopted to describe the joint behaviour. The mechanical properties of the joint, including the elastic stiffness, friction, cohesion, tensile strength and dilatancy, were fully considered in this model. A strain softening characteristic appears when the shear or tensile failure parameters decrease. This joint model is especially suitable for underground excavation simulations. The constitutive behaviour of the contact is shown in Fig. 8^31^. The contact force was divided into normal stress and shear stress. The stress-displacement relation was assumed to be linear in the normal direction, and the normal stiffness can be expressed as:

3 $$\Delta \sigma_{{\text{n}}} = - k_{{\text{n}}} \Delta u_{{\text{n}}}$$

where $\Delta \sigma_{{\text{n}}}$ is the increment of the effective normal stress, $\Delta u_{{\text{n}}}$ is the increment of the normal displacement, and $k_{{\text{n}}}$ is the normal stiffness.

Tensile failure occurs in the contact when $\sigma_{{\text{n}}}$ equals 0; at this time, the normal stress exceeds the tensile strength of the contact.

In the shear direction of the contact, the stress-displacement relation can be expressed at two stages. If $\left| {\tau_{{\text{s}}} } \right| \le c + \sigma_{{\text{n}}} \tan \varphi$, then

4 $$\tau_{{\text{s}}} { = }k_{s} \Delta u_{{\text{s}}}^{{\text{e}}}$$

If $$\left| {\tau_{{\text{s}}} } \right| \ge \tau_{\max }$$, then

5 $$\Delta \tau_{{\text{s}}} {\text{ = sign(}}\Delta u_{{\text{s}}} {)}\tau_{{{\text{max}}}}$$

where $k_{{\text{s}}}$ is the shear stiffness, $\tau_{{{\text{max}}}}$ is the shear strength, $\Delta u_{{\text{s}}}^{{\text{e}}}$ is the increment of the elastic shear displacement, and $\Delta u_{{\text{s}}}$ is the increment of the total shear displacement.

#### Modelling

1. Model setup

**Figure 9:**
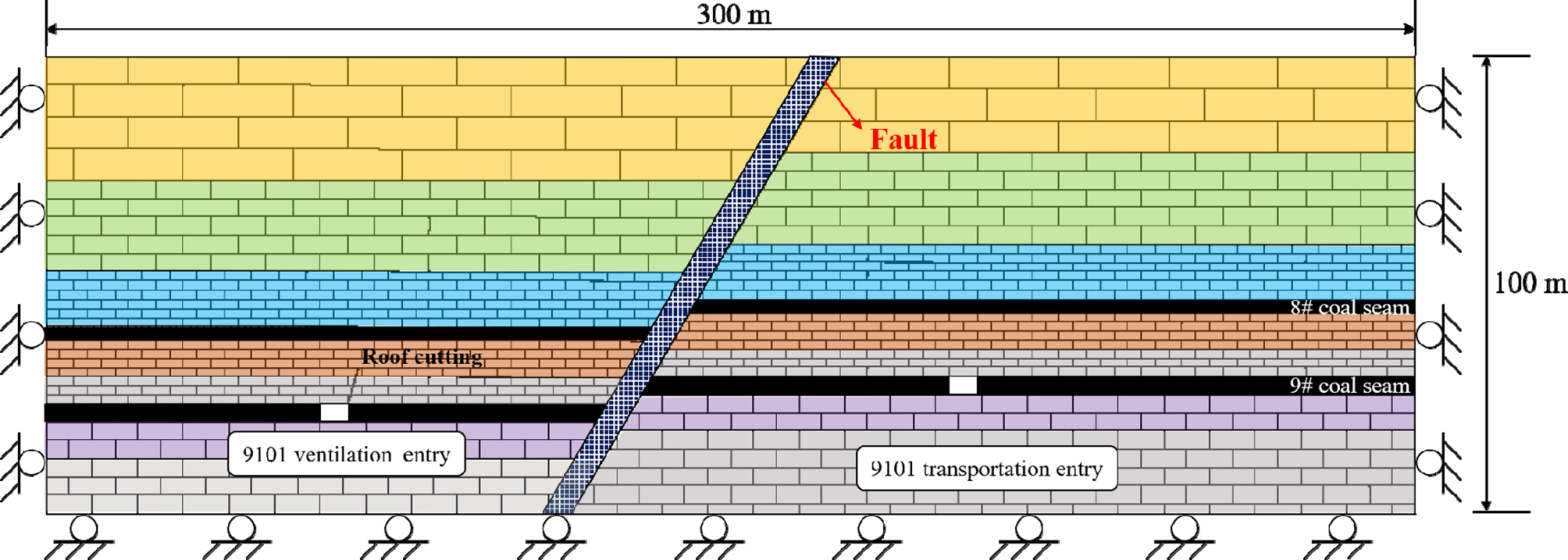
Basic model for the DEM simulation.

According to the geological conditions of the test site, as the mining face advances, the starting position of the formed roadway shifts from near the fault to away from the fault, finally approaching the remaining pillar of the 8# coal seam. To simulate the whole process, a series of calculation models for different roadway formation conditions were established. The dimensions for the two-dimensional models were 300 m long and 100 m high, based on the geological column. The displacement boundaries of the models were set as roller boundaries along the sides and a pinned boundary along the bottom, as shown in Fig. 9. The floor of the 9# coal seam was post stone, the floor of the 8# coal seam was sandy mudstone, and the thickness of the rock stratum between the 8# and 9# coal seams was 14 m. Roof cutting was simulated using two parallel cracks. The distance between the two cracks was 50 mm, which is the same as the diameter of the split blasting hole. The fracture zone of the fault was considered to be composed of low modulus materials, and interfaces were added along the fault sides. The normal stiffness of the discontinuity structure is 50 GPa/m, shear stiffness is 5 GPa/m, cohesion and tensile strength are both 0.

(2) Calibration of modelling parameters

In the simulation, the Mohr–Coulomb constitutive model was applied to describe the rock block behaviour, and the Coulomb slip joint model with surfaces in contact was used to describe the joint behaviour. The parameters used in the simulation were calibrated based on a rock mechanical test in the laboratory. The direct back analysis method, which is based on repetition and minimization of the error function, was used to determine reasonable parameters. The deformation of the roadway surroundings was selected as an indicator. The objective function can be expressed as:

6 $$E_{r} = \frac{{\sum {(u_{k} - u_{k}^{*} )^{2} } }}{{\sum {u_{k}^{*} } }}$$

where $E_{r}$ is the error value, $u_{k}$ is the calculated displacement in the simulation, and $u_{k}^{*}$ is the measured value in the field.

Typical analysis is performed by selecting an initial value; the convergence of the objective function is then calculated by Eq. (6); and the computational step ends when it reaches the actual convergence value^32^. Based on the above, the results of the direct back analysis method on the 9101 ventilation roadway were applied to perform the back analysis.

**Figure 10:**
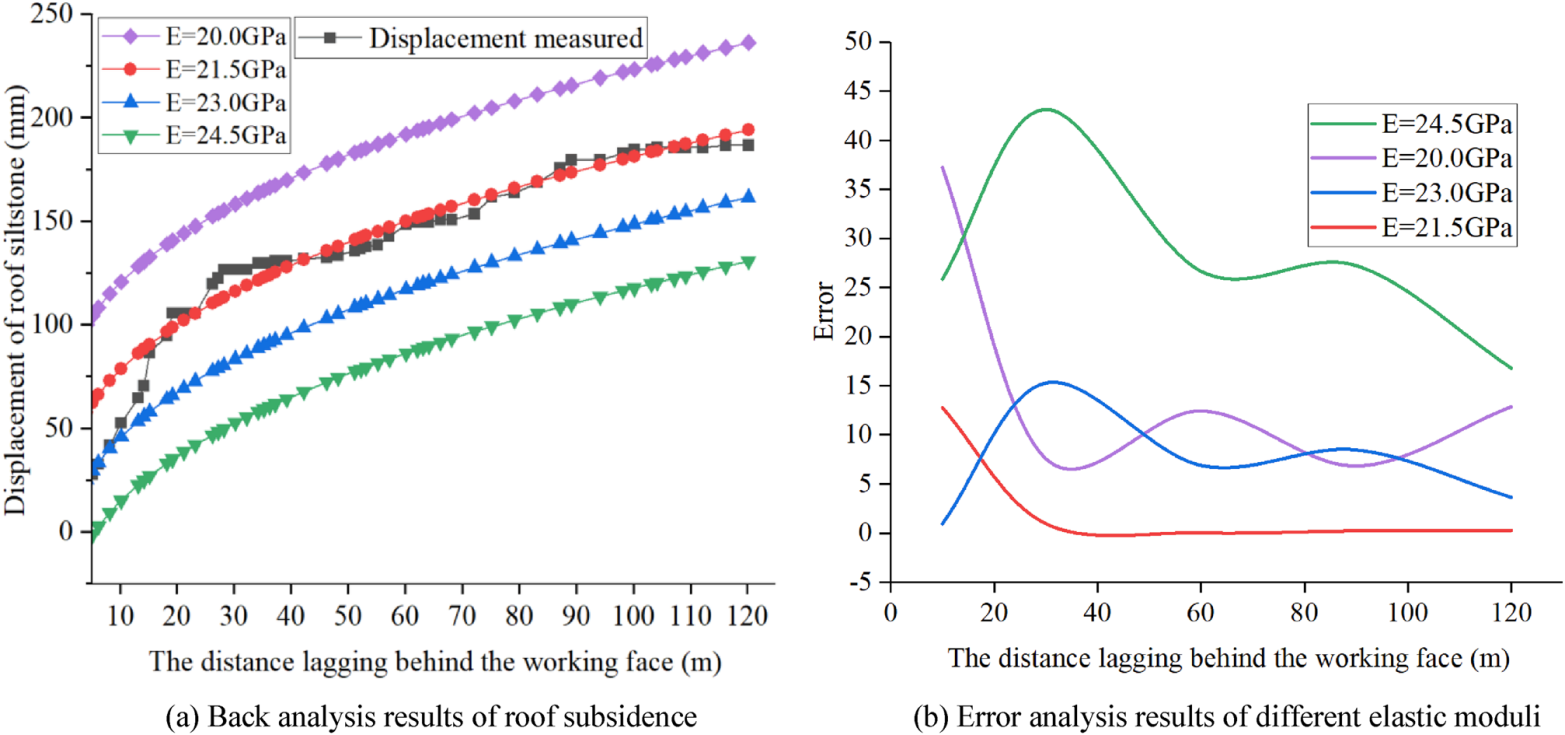
Calibration of elastic modulus of post stone in floor.

The calibration process of the elastic modulus is shown in Fig. 10. Through an analogous analysis, it was determined that the elastic modulus of the post stone in the coal seam floor was between 20.0 GPa and 25.0 GPa, and then a series of simulation calculations were carried out. The simulation results (Fig. 10a) show that when E = 21.5 GPa, the roof subsidence has a strong correlation with the measured value, and when Formula (4) was used to calculate the error analysis results (Fig. 10b), the error value was the smallest when E = 21.5 GPa. Therefore, based on the back analysis, E = 21.5 GPa was a reasonable estimate of the elastic modulus of the post stone in the floor. The other parameters were obtained by the same method or calculated theoretically using Eqs. (7) and (8). The final parameters of blocks and contacts for the strata are summarized in Table 1.

**Table 1:** Mechanical parameters of the rock strata for the numerical model.

Rock strata	Density (kg/m^3^)	Elasticity modulus (GPa)	Friction angle (deg)	Cohesion (MPa)	Tensile strength (MPa)	$k_{n}$(GPa/m)	$k_{s}$(GPa/m)
Overlying strata	2500	17.2	30	1.8	0.71	112.9	45.2
Limestone	2620	27.1	35	3.2	1.40	218.5	84.0
8# coal	1500	3.80	21	0.9	0.35	89.2	35.7
Sandy mudstone	2500	10.1	30	2.4	1.20	123.1	49.2
Mudstone	1900	13.5	28	2.4	1.30	79.20	31.7
9# coal	1500	3.80	21	0.9	0.35	89.20	35.7
Post stone	2550	21.5	30	2.1	0.80	217.9	87.2
Mudstone	1900	13.5	28	2.4	1.30	79.20	31.7

The normal stiffness ($k_{n}$) and shear stiffness ($k_{s}$) of the contacts were obtained by using the following formulas:

7 $$k_{{\text{n}}} = 10\left[ {\frac{{K + \frac{3}{4}G}}{{\Delta z_{\min } }}} \right]$$

8 $$k_{{\text{s}}} = 0.4k_{{\text{n}}}$$

where *K* is the bulk modulus, calculated by $K = E/3(1 - 2\mu )$, *G* is the shear modulus of the blocks, calculated by $G = E/2(1 + \mu )$, and $\Delta z_{\min }$ is the smallest width of the zone adjacent to the contact in the normal direction.

## Strata behaviour analysis during the roadway formation

Compared with previous studies on the ARFRC method, the unique feature of this study is the existence of faults and goafs. Previous studies have shown that the stress distribution and deformation of the roadway surroundings are the main factors that reflect the stability of the roadway surroundings. In this study, the influence of faults and goafs on the stability of roadways and working panels was studied using field monitoring data. The influence of mining and roadway formation on the stress distribution and overburden strata movement around the geologic structure was analysed with DEM simulations.

### Roof loads in the active working face

**Figure 11:**
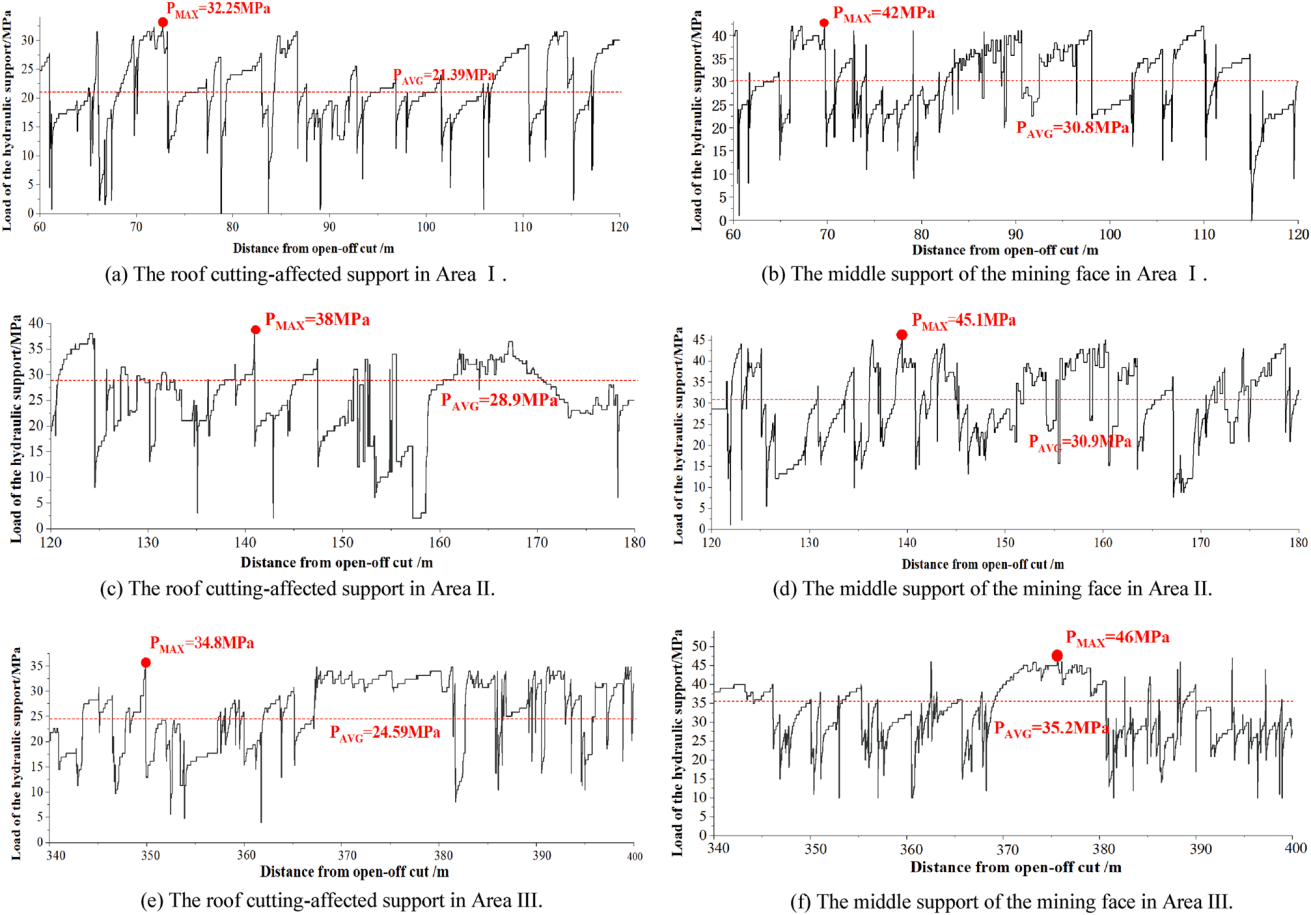
Variation in the roof loads in the active mining face for different test sections.

Before mining, the roof of the mining face and the gob are a whole. As the gob roof caves, the overlying strata of the gob moves and can influence the loads on the support. In a similar way, the roof of the formed roadway is also affected by the caving of the gob roof and movement of the overlying strata. Therefore, the roof loads of the support, movement of the roadway surroundings and caving states of the overburden strata are related. In the field test, two typical hydraulic supports where are 5 m in the roof cutting-affected area and 100 m in the middle of the mine face are selected to monitor the roof loads in the mining face. Both hydraulic supports had online pressure monitoring sensors installed. According to the geological characteristics of the study site, the formed roadway was affected by the above gob from mining positions of 60 m to 120 m (Area I). From mining positions of 120 m to 180 m (Area II), the formed roadway was affected by the fault, and from mining positions of 340 m to 400 m (Area III), the formed roadway was affected by the 8# coal pillar. In these three typical areas, the loads of two typical hydraulic supports were monitored, as shown in Fig. 11.

In Area I, the maximum value of the support load in the roof cutting-affected area was 32.25 MPa, and the average value was 21.39 MPa. However, in the middle of the active mining face, the maximum and average support loads were 42 MPa and 30.8 MPa, respectively, which were considerably larger than those in the roof cutting-affected area. This phenomenon is mainly caused by the collapsed structure of the overlying strata above the working face. Another factor is the influence of roof cutting. Within the roof cutting-affected area, the caved roof rock is bulkier and can fully support the main roof behind the hydraulic supports. Because the rotated deformation of the main roof is small, the roof pressure acting on the hydraulic supports is reduced.

Area II was affected by the fault. The average load of the support in the roof cutting-affected area was 28.9 MPa, which was 35% greater than the support pressure at the same position in Area I. The load distribution indicates that the movement of the overlying strata is more intense in the fault-affected area. Because the fault is diagonal to the working face and the middle of the mining face is not in the fault area, the peak and average loads of the supports in the middle of the mine face in Areas I and II are nearly the same.

Area III was affected by the 8# coal pillar of the goaf. In this area, the loads of the hydraulic support were particularly large from 370 to 380 m. There were goafs on both sides of the 8# coal pillar, and the coal pillar was a stress-concentrated area. When the mining face was below the coal pillar, the high stress on the coal pillar was transmitted to the hydraulic supports. The average loads of the supports were approximately 15% larger than those in the same position of Area I. The retained coal pillar also has an influence on mining, but it has a smaller impact than the fault.

**Figure 12:**
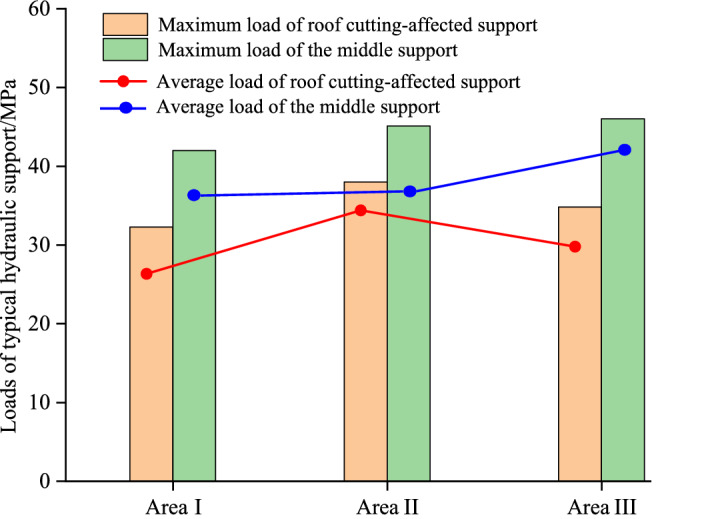
Statistics of the maximum and average roof loads in different test areas.

The maximum and average loads of the two typical hydraulic supports in these three areas are summarized in Fig. 12. The figure shows that the support load in the fault-affected area is considerably less than another one, indicating that the pressure transmission of the roof strata can be effectively reduced after cutting the roof strata. In addition, in the fault-affected area, the maximum load and average load of the support are greater than those in the same position in Area I, increasing by approximately 35%. In the coal pillar-affected area, the average load of the support was approximately 15% larger than that at the same position in Area I. This shows that, under the same geological conditions, the stability of the formed roadway was most affected by faults.

### Movement of the roadway surroundings

**Figure 13:**
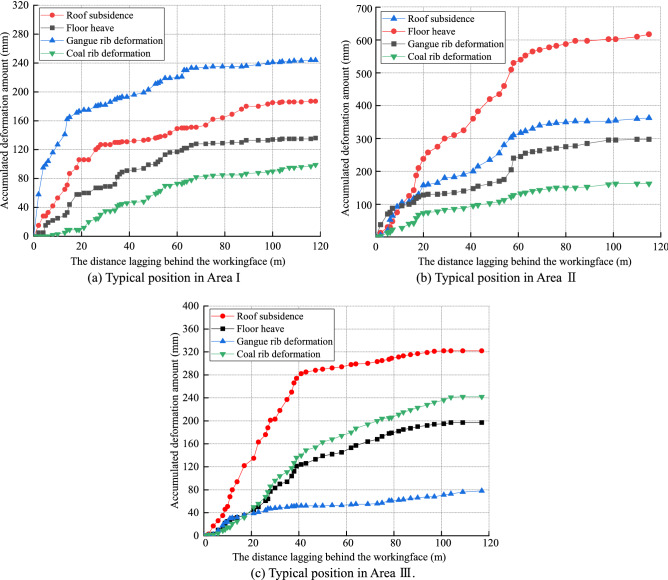
Deformation characteristics of the roadway surroundings in the three areas.

Deformations of the roadway surroundings were monitored in the formed roadway to investigate the influence of gobs, faults and coal pillars on the failure of the formed roadway. Specifically, in Areas I, II and III, the deformations of the roof, floor, gangue rib and coal rib were recorded at typical monitoring points, and the roadway deformation at the typical monitoring positions of the three areas was essentially 120 m behind the working face and tended to be stable, as shown in Fig. 13. When the formed roadway was below the goaf, the overall deformation of the roadway surroundings was small. The largest and smallest deformations of the roadway sides were observed in the gangue rib and the coal rib. The stabilized roof-to-floor convergence was 335 mm. When the formed roadway was in the fault-affected area, the overall deformation of the roadway surroundings significantly increased. The roof sagged by 625 mm, which was the largest among the four sides. The second largest deformation of the roadway sides was observed in the gangue rib. This phenomenon indicates that the movement of the fault has a significant influence on the overlying strata of the formed roadway. However, in the coal pillar-affected area, the deformation of the coal rib and floor heave were relatively large. The coal pillar was a stress-concentrated area, and the hardness coefficients of the coal and floor were small, resulting in large deformations of the two sides. Different areas exhibit different stress characteristics, which results in distinct deformation characteristics in the surroundings of the formed roadway.

### Caving states of the overburden strata

**Figure 14:**
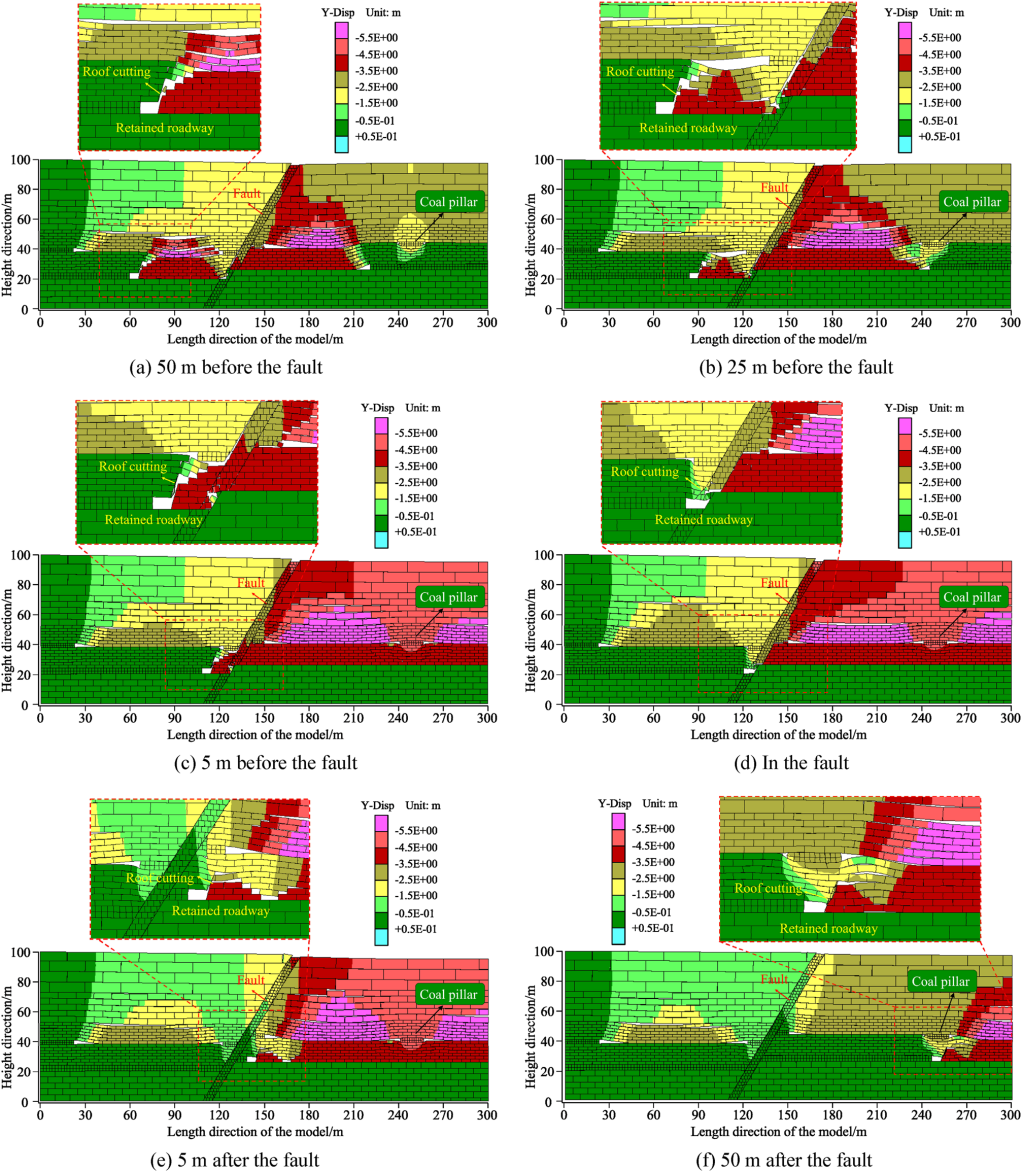
Evolutionary process of the roadway surroundings under different mining and roadway formation conditions.

As the working face advances forwards, the formed roadway gradually approaches the fault. To explore the caving and movement characteristics of the roadway surroundings while crossing the fault, numerical modelling of the roadway formation at different positions from the fault was performed. Roadway formations were simulated at positions of − 50 m, − 25 m, − 5 m, 0 m, 5 m, and 50 m from the fault. A vertical displacement field with an overburden structure is shown in Fig. 14.

When the formed roadway was 50 m ahead of the fault, the mining face was divided into two similar parts by the fault. The roof of the working face in the hanging wall caved in and became the gangue rib of the formed roadway. The 8# coal seam roof collapsed and pressed on the 9# coal seam roof. The overlying strata of the working face in the footwall of the fault caved along the fault surface, and the vertical displacement was largest at 185 m (middle of the footwall working face). In this scheme, the formed roadway was far from the fault, and the deformation of the roadway surroundings was small. When the formed roadway was 25 m ahead of the fault, the 9# coal seam roof fully caved in, forming a suspended roof structure above the 8# coal seam in the footwall. The largest vertical displacement in the footwall working face was observed at 200 m. In the third scheme, most of the working face was in the footwall of the fault. The deformation of the roadway surroundings began to noticeably increase, and the stabilized deformation of the roadway roof was 550 mm. When the formed roadway was in the fault fracture zone, the deformation of the roadway surroundings was the largest. Some of the roadway roof was a fault fracture zone. Due to the concentration of tectonic stress in the fault fracture zone, the roof was unstable. Thus, the support strength needs to be increased to address the high stress around the roadway.

When the formed roadway crosses the fault (see Fig. 14e), the entire mining face is in the footwall of the fault. The roof and coal rib diagonally support the fault surface, and the caved gangues at the gob are in close contact with the roadway roof. Under this condition, a stable diagonal structure was formed, reducing the deformation of the roadway roof. As the formed roadway moved away from the fault, the influence of the fault on the stability of the formed roadway gradually weakened. However, when the formed roadway was below the 8# coal pillar, the deformation of the roadway roof began to increase, reaching a maximum deformation of 320 mm. The simulated results agree well with the measured results.

**Figure 15:**
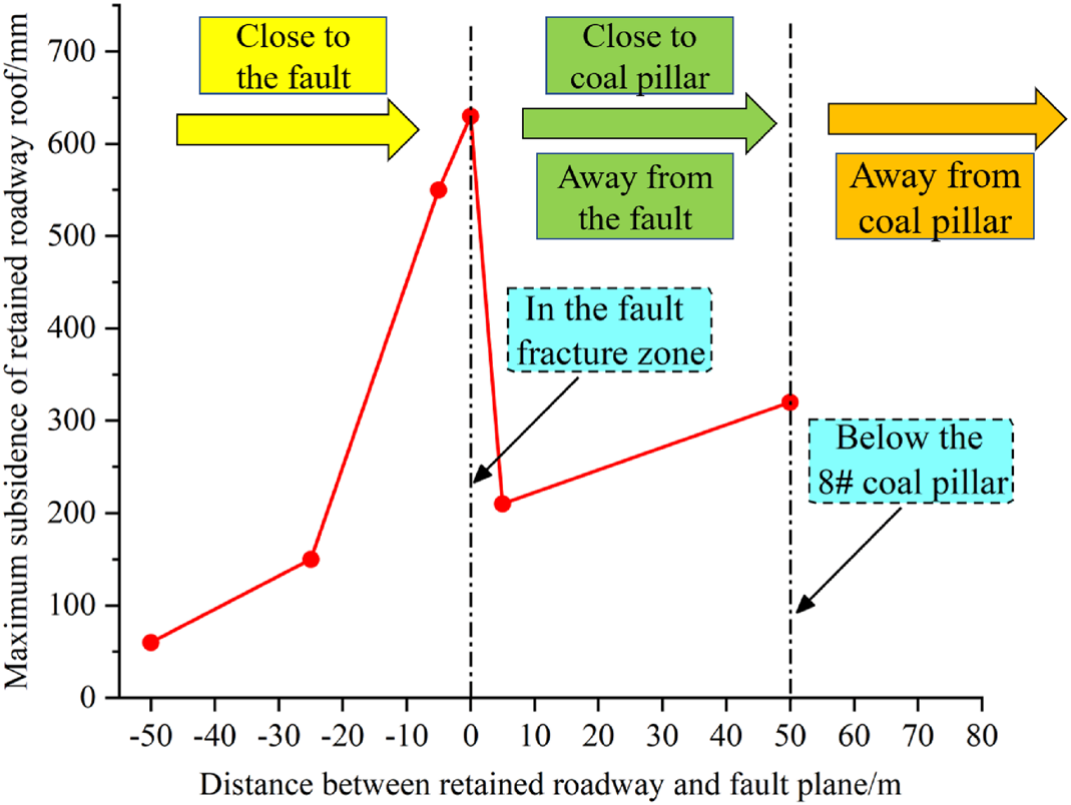
Summary of the maximum subsidence of the formed roadway roof under different mining positions.

The maximum subsidence values of the roadway roof for different mining positions are summarized in Fig. 15. The maximum subsidence of the roof gradually increased when the formed roadway position moved closer to the fault, indicating that the stability of the formed roadway was reduced due to fault activation. When the formed roadway was located in the fault fracture zone, the roadway roof subsidence was the largest. In this case, the support strength needs to be increased to ensure that the roadway roof deformation remains within a reasonable range. When the position of the formed roadway moved away from the fault, the subsidence of the roadway roof decreased, indicating that the influence of the tectonic stress on the roadway gradually decreased after fault activation. When the formed roadway gradually approached the 8# coal pillar, due to the high stress concentration under the coal pillar, the maximum subsidence of the roadway roof increased, and the stability of the reserved roadway decreased. However, it is worth noting that the subsidence of the roof was much greater when the formed roadway was located in the fault fracture zone than that when it was located below the 8# coal pillar.

### Behaviour of the fault plane

In coal fields, geologic structures such as faults are widely distributed. The tectonic stress induced by geological anomalies and the mining stress induced by dynamic disturbances are often coupled, which causes the stress field to be more complicated. In our study, the interaction between geological anomalies and roadway formation was systematically studied. It is well known that the fault geologic structure greatly influences the application of the ARFRC method. In contrast, how do mining and roadway formation affect fault activation? In this section, the responses of the fault plane to the mining and roadway formation process are analysed.

1. Normal stress evolution

**Figure 16:**
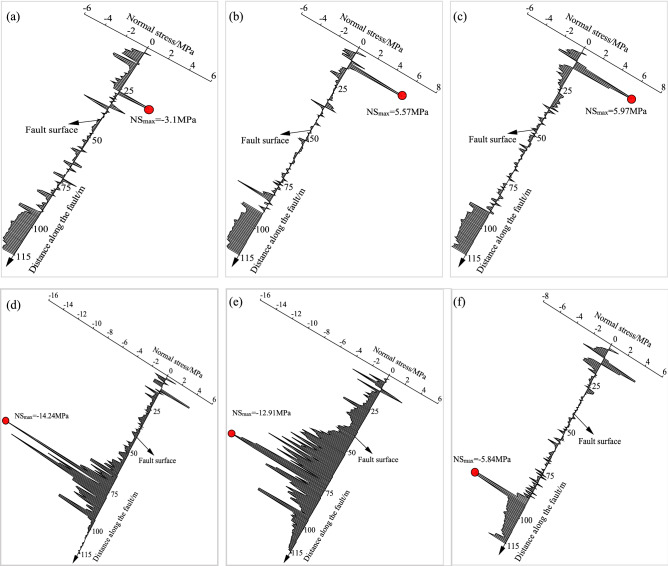
Evolutionary process of the normal stress along the fault plane under different roadway formation positions.

Normal stress is the force perpendicular to the fault plane. The normal stress along the fault was recorded under different mining and roadway formation positions (− 50 m, − 25 m, − 5 m, 0 m, 5 m, and 50 m from the fault), as shown in Fig. 16. When the formed roadway was at the hanging wall of the fault, the normal stress was larger and mainly distributed in the upper and lower parts of the fault plane, with the largest normal stress observed in the upper fault plane and pointing in the positive direction of the *x*-axis. Under these conditions, the mining face was divided by the fault. Due to coal body mining, the overburden strata of the working face were discrete, so the contact force in the middle of the fault surface was small. When the formed roadway was 50 m before the fault, the maximum normal stress was 3.1 MPa. As the formed roadway gradually approached the fault, the maximum normal stress increased to 5.97 MPa (Fig. 16c). When the formed roadway was in or after the fault fracture zone, the stress distribution was clearly affected by the lateral abutment pressure of the mining face. In the last three roadway formation conditions, the main stress section was the lower part of the fault plane. The maximum normal stress was 14.24 MPa, pointing in the negative direction of the *x*-axis. As the formed roadway moved away from the fault, the stress on the fault plane gradually decreased.

(2) Shear stress evolution

**Figure 17:**
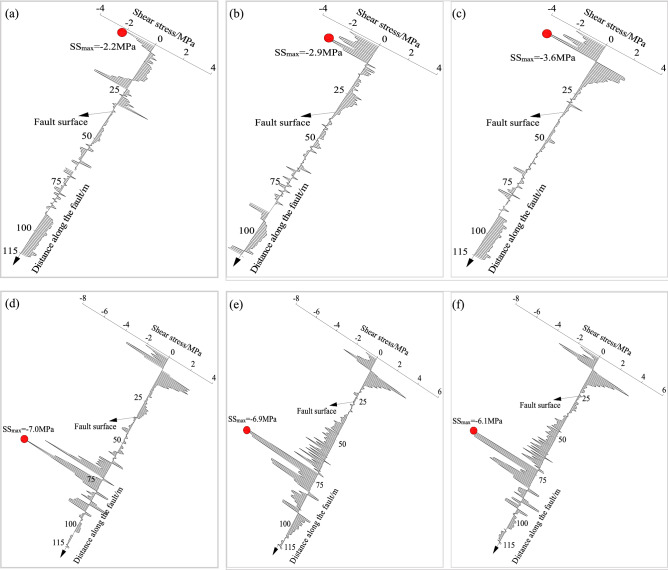
Evolutionary process of the shear stress along the fault plane under different roadway formation positions.

Shear stress refers to the force parallel to the fault plane. The distributions of the shear stress along the fault under different roadway formation conditions (− 50 m, − 25 m, − 5 m, 0 m, 5 m, and 50 m from the fault) are shown in Fig. 17. When the formed roadway was in the hanging wall of the fault, the overall shear stress was small, and shear stress with larger values occurred mainly in the upper part of the fault plane. As the formed roadway gradually approached the fault, the shear stress increased. The maximum value of the shear stress was 3.6 MPa when the formed roadway was in the hanging wall. When the formed roadway was in the fault fracture zone or footwall of the fault, the largest stress occurred in the lower part of the fault, approximately 75 m from the top boundary of the model. The overall shear stress was larger than when the formed roadway was in the hanging wall, with a maximum shear stress of 7.0 MPa. When the entire mining face was in the footwall of the fault, the overlying strata moved in the direction of the mined-out area, which, when combined with the effects of the lateral abutment pressure, caused a downwards shear stress to be formed on the fault plane.

(3) Displacement evolution

**Figure 18:**
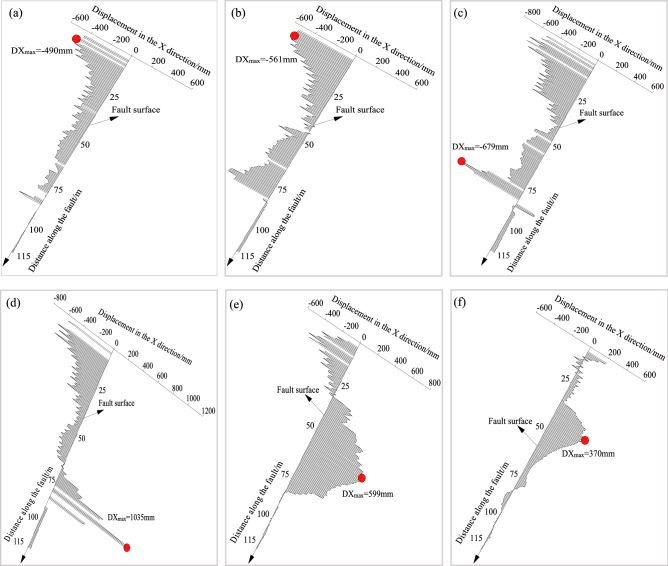
Evolutionary process of the horizontal displacement along the fault plane under different roadway formation positions.

The vertical displacement of the overlying strata has been discussed previously. In this section, the horizontal displacement along the fault plane was emphatically analysed, as shown in Fig. 18. When the formed roadway was in the hanging wall of the fault, the mining room in the hanging wall bent the overlying rock strata, causing the horizontal displacement near the top of the model to be large and in the negative direction of the *x*-axis. The horizontal displacement increased as the formed roadway approached the fault, reaching a maximum value of 679 mm. When the formed roadway was in the fault fracture zone, the largest horizontal deformation of 1035 mm can be seen in Fig. 18d. As the formed roadway moved to the footwall of the fault, the main deformation area changed to the caving range of the working face, pointing in the positive direction of the *x*-axis (Fig. 18e and f).

In general, the stress and displacement characteristics of the roadway surroundings under typical geological conditions and the influence of roadway formation and mining on the geological structure were obtained. The stress and displacement variation in the fault plane can reflect the stability of the formed roadway. It should be noted that the fault affecting roadway formation in this study was a normal fault, and the roadway formation process occurred from the hanging wall to the footwall. For a reverse fault in a different mining direction, the strata behaviour and roadway formation laws need to be further investigated.

## Stability control of the formed roadway

From the experimental and simulated results, we can conclude that when the working face is mined under different geological conditions, the formed roadway surroundings exhibit different behaviours in terms of stress and displacement. Under the influence of a fault influenced longwall goaf, the stress of the roadway surroundings was significantly higher than that under conventional geological conditions. To ensure the stability of the formed roadway, the support needs to be upgraded.

**Figure 19:**
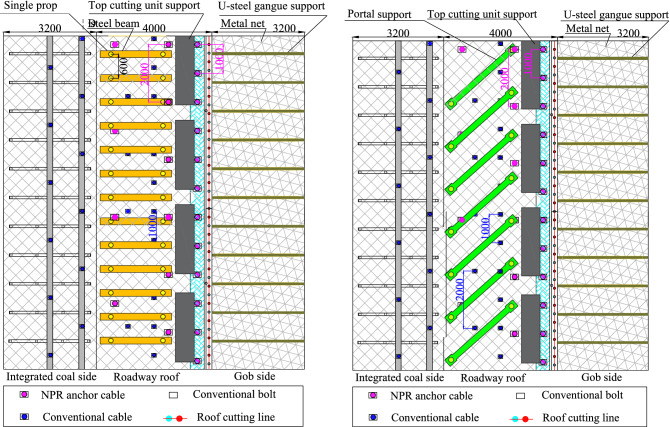
Support design for the formed roadway under the influence of a fault and above a coal pillar.

To ensure the stability of formed roadway, new control techniques based on the deformation and stress characteristics of the roadway surroundings were proposed. In the fault-affected area, the formed roadway passes through the normal fault from hanging wall to foot wall. Owing to the influence of fault fracture zone, there is additional room in the roof. In the field, wooden poles were first used to fill the roof. The top cutting unit support and portal support were then used as temporary supports with a support range of 120 m. The top cutting unit support was composed of two columns with high support resistance. The working resistance of the top cutting unit support was 4000 kN, and the initial support resistance was 3090 kN. The longitudinal deflection angle of the top beam was ± 15°, and the transverse deflection angle was ± 5°. The portal support was composed of two single hydraulic props that were articulated with the roof beam. The support height of the portal support ranged between 2200 and 4100 mm, and the support resistance was 2040 kN. As shown in Fig. 19a, the top cutting unit supports were placed on the roof cutting side. The distance between two adjacent top cutting unit supports was 0.5 m. The portal supports were placed on the integrated coal side. The row spacing of the portal supports was 2 m.

Within the influence range of the 8# coal pillar, the stress of the formed roadway surroundings increases to a certain extent, but the deformation caused by the stress is smaller than that in the fault-affected area. Therefore, we proposed another support scheme for roadway formed in the influence range of the 8# coal pillar. In this area, the portal support was replaced with two rows of single hydraulic props with a π beam. The temporary support range in this area is 80 m. The distance between two rows of single hydraulic props was 1000 mm. The design plan is shown in Fig. 19b.

**Figure 20:**
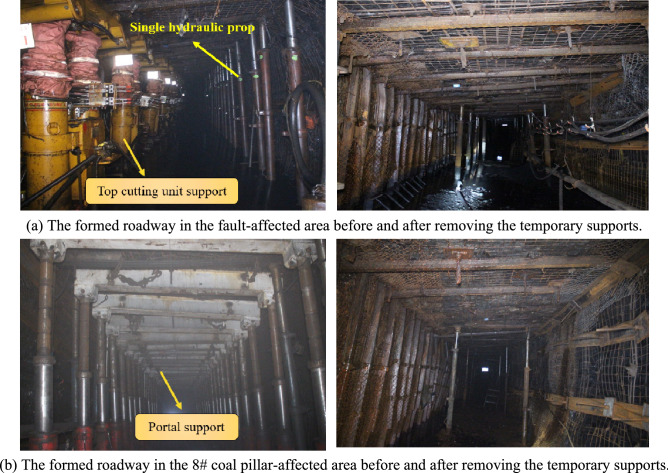
Application effects of the ARFRC method in the affected areas of the geologic faults and the 8# coal pillar.

The formation effects of the roadway in the field application are shown in Fig. 20. The deformations of the surrounding rock in the formed roadway in the fault-affected area and the coal pillar-affected area were effectively controlled using comprehensive control measures, demonstrating the reliability and rationality of the proposed support techniques.

## Conclusions

Field experiments and numerical simulations were conducted to study the strata behaviour and failure mechanism of the formed roadway in the ARFRC mining method below a fault influenced longwall goaf. The main conclusions of this study are summarized below.

1. The hydraulic support loads were monitored to investigate the stress distribution of the overlying strata above the working face. When the formed roadway was located in the fault-affected area, the roof loads of the working face around the formed roadway were appropriately 35% larger than those above the goaf. The side loads of the working face were greater than the middle loads of the working face. The collected data indicated that the existence of a fault causes the overlying strata to move, and the support loads were considerably affected by the fault. The coal pillar above the mining face increased the loads of the hydraulic supports by 15%. Thus, for a close coal seam group, it is recommended to use the ARFRC nonpillar mining method on the upper coal seam.
2. In the fault- and coal pillar-affected areas, the deformations of the roadway surroundings increased. Roof sagging was the largest in the fault-affected area, and deformations of the integrated coal and floor were larger in the coal pillar-affected area. In different geological areas under the goaf, the stress distribution of the roadway surroundings leads to different deformation characteristics. The numerical modelling results indicated that when the formed roadway was in the hanging wall and approached the fault plane, the vertical displacement of the roof gradually increased, with the largest deformation appearing when the formed roadway was in the fault fracture zone. After crossing the fault, the vertical displacement of the roadway surroundings noticeably decreased.
3. The evolution laws of the normal and shear stresses along the fault plane during the mining and roadway formation processes were studied with DEM modelling. When the formed roadway was in the hanging wall, the area with the maximum normal stress was in the overlying strata of the mining face, and the direction of the maximum normal stress pointed towards the footwall. As the formed roadway approached the fault plane, the normal stress gradually increased. In terms of shear stress, the maximum shear stress in the fault plane was downwards, and the value of the shear stress decreased as the formed roadway moved away from the fault.
4. Practice showed that conventional supports cannot meet the control requirements of the roadway surroundings formed under the influence of fault influenced longwall goafs. New ARFRC support techniques characterized by composite temporary supports were proposed and specific parameters were designed under special geological conditions according to the obtained strata behaviour. The satisfactory roadway formation effects proved the reliability and rationality of the techniques.

## Data Availability

All data used to support the findings of this study are available from the corresponding author upon request.
